# Antibiotic susceptibility of coagulase-negative staphylococci isolated from very low birth weight babies: comprehensive comparisons of bacteria at different stages of biofilm formation

**DOI:** 10.1186/1476-0711-9-16

**Published:** 2010-05-27

**Authors:** Yue Qu, Andrew J Daley, Taghrid S Istivan, Suzanne M Garland, Margaret A Deighton

**Affiliations:** 1 School of Applied Sciences, RMIT University, Australia; 2 Department of Microbiology, Royal Children's Hospital, Victoria, Australia; 3 Department of Obstetrics and Gynaecology, University of Melbourne, Australia; 4 Department of Microbiology and Infectious Diseases, Royal Women's Hospital, Victoria, Australia

## Abstract

**Background:**

Coagulase-negative staphylococci are major causes of bloodstream infections in very low birth weight babies cared for in Neonatal Intensive Care Units. The virulence of these bacteria is mainly due to their ability to form biofilms on indwelling medical devices. Biofilm-related infections often fail to respond to antibiotic chemotherapy guided by conventional antibiotic susceptibility tests.

**Methods:**

Coagulase-negative staphylococcal blood culture isolates were grown in different phases relevant to biofilm formation: planktonic cells at mid-log phase, planktonic cells at stationary phase, adherent monolayers and mature biofilms and their susceptibilities to conventional antibiotics were assessed. The effects of oxacillin, gentamicin, and vancomycin on preformed biofilms, at the highest achievable serum concentrations were examined. Epifluorescence microscopy and confocal laser scanning microscopy in combination with bacterial viability staining and polysaccharide staining were used to confirm the stimulatory effects of antibiotics on biofilms.

**Results:**

Most coagulase-negative staphylococcal clinical isolates were resistant to penicillin G (100%), gentamicin (83.3%) and oxacillin (91.7%) and susceptible to vancomycin (100%), ciprofloxacin (100%), and rifampicin (79.2%). Bacteria grown as adherent monolayers showed similar susceptibilities to their planktonic counterparts at mid-log phase. Isolates in a biofilm growth mode were more resistant to antibiotics than both planktonic cultures at mid-log phase and adherent monolayers; however they were equally resistant or less resistant than planktonic cells at stationary phase. Moreover, for some cell-wall active antibiotics, concentrations higher than conventional MICs were required to prevent the establishment of planktonic cultures from biofilms. Finally, the biofilm-growth of two *S. capitis* isolates could be enhanced by oxacillin at the highest achievable serum concentration.

**Conclusion:**

We conclude that the resistance of coagulase-negative staphylococci to multiple antibiotics initially remain similar when the bacteria shift from a planktonic growth mode into an early attached mode, then increase significantly as the adherent mode further develops. Furthermore, preformed biofilms of some CoNS are enhanced by oxacillin in a dose-dependent manner.

## Background

Coagulase-negative staphylococci (CoNS), predominantly *Staphylococcus epidermidis*, are the most common causative agents of neonatal sepsis [1-3], a condition which has been related to significant morbidity and mortality in neonatal intensive care units (NICUs) [2]. The presence of a central venous catheter in very low birth weight (VLBW) babies (< 1500 g) is a significant risk factor for CoNS-associated neonatal sepsis [3-5].

The pathogenesis of CoNS infections depends on their ability to form biofilms on polymer surfaces [6-8]. After initial contact and attachment to a surface, the bacterial cells become embedded in an extracellular polymer substance, which is composed of water, exopolysaccharide, extracellular DNA, proteins, and lipids [9,10]. Once grown as a biofilm, the embedded bacteria are protected from various physical, chemical and biological stresses [11,12]. Specifically, biofilms formed by CoNS clinical isolates possess an extremely high level of tolerance to multiple antibiotics [13,14]. Antibiotic resistance of CoNS biofilms is likely to have multiple causes including failure of antibiotics to reach the extracellular polymer substance embedded biofilm cells, a micro-environment unfavourable to antimicrobial activity, slow bacterial growth, activation of stress responses within biofilms, phenotypically resistant persister cells, and genotypically resistant cells selected by antibiotic exposure in biofilms [15,16].

Antibiotic treatment based on standard in vitro susceptibility tests designed for planktonic bacteria, such as the Clinical and Laboratory Standards Institute (CLSI) method, fails to clear biofilm-related CoNS infections. It would therefore seem more appropriate to base the treatment on susceptibility profiles of CoNS grown as biofilms. Numerous studies have been conducted to compare the antibiotic susceptibilities of biofilm-grown bacteria and their planktonic counterparts, but different results were reported [17,19,20]. Most studies found that biofilm-grown bacteria were 100-1000 times more resistant than their planktonic counterparts [18-20], however, Spoering and Lewis (2001) stated that bacteria grown as biofilms and planktonic cells had similar antibiotic susceptibilities [17]. This inconsistency could be explained by the differences in methodologies used. Some studies [19-21] compared susceptibilities of planktonic cultures at ~5 × 10^5 ^CFU/ml with mature biofilms, which contain ~10^10-11^ CFU/ml of individual bacteria. It has been shown that antibiotic resistance of *Staphylococcus* spp. is inoculum size dependent [22]. Others [21,23,24] compared standard MICs that target growth inhibition, with minimum biofilm eradication concentrations (MBECs) that target complete bacterial killing.

Distinct bacterial activities are found in different phases of biofilm-related infections. In the febrile phase, large numbers of planktonic cells originating from the biofilms are present in the surrounding tissues or the bloodstream, and are responsible for the acute inflammatory response. This transitory phase is followed by a prolonged chronic phase, in which biofilm bacteria are more likely to remain static and cause milder clinical symptoms [12,25]. When immune competence is weakened or antimicrobial stresses subside, biofilms can be re-activated and cells can again detach and colonize other sites to cause new symptoms locally or remotely [26,27].

The biofilm MIC is an in vitro guide to antibiotic use to prevent biofilms from releasing planktonic cells and establishing the febrile phase of infections [23,24]. Ceri et al. (2001) suggested that conventional CLSI MICs could be used to represent biofilm MICs to inform antibiotic treatment, as these two values were generally similar [23]. However, other studies [24,28] have found that biofilm MICs of some antibiotics are significantly higher than the CLSI MICs. In the chronic phase of biofilm infections which are characterised by less active dispersal, the MBEC, which targets complete bacterial killing would be more meaningful than the biofilm MIC in predicting the clinical response [20,23]. The MBEC is an impractical goal when conventional antibiotics are used in vitro [3,29], but the biofilm MBC, which targets 99.9% killing of biofilm-grown bacteria, may be achievable.

Biofilms produced by CoNS consist principally of a polysaccharide intercellular adhesin (PIA), which is encoded by the *icaADBC* operon [8]. Tetracycline, erythromycinand quinupristin-dalfopristin,at subinhibitory concentrations, were reported to enhance CoNS biofilm formation by stimulating *ica *expression, whereas no positive effect was found for penicillin,oxacillin, chloramphenicol, clindamycin, gentamicin, ofloxacin,vancomycin, or teicoplanin [30-32]. Most previous studies examined the effect of antibiotics on biofilm formation of planktonic bacteria [30-32], whereas established mature biofilms are more commonly encountered in infections of VLBW infants in NICUs. In addition, those studies examined the effect of antibiotics at subinhibitory concentrations; however concentrations achievable in serum would be more clinically relevant.

The objectives of this study were to (i) compare the antibiotic susceptibility of CoNS in different stages of biofilm formation (ii) assess the contribution of polysaccharide to resistance of biofilm-grown bacteria to antibiotics (iii) determine any stimulatory effects of the first line antibiotics used in NICUs on preformed CoNS biofilms.

## Methods

### Staphylococcal isolates

Twenty-four CoNS isolates from blood cultures of VLBW newborns receiving treatment at the Royal Women's Hospital (RWH) NICU were examined to obtain an overall view of the antibiotic susceptibility patterns of strains circulating in the unit and to determine the effect of antibiotics at serum achievable concentrations on preformed biofilms (Table 1) [33]. These isolates were identified previously to species level, examined for their ability to produce biofilms and classified as invasive isolates or probable contaminants [33] according to the definition of sepsis in CDC-NNIS (United States Centre for Disease Control and Prevention National Nosocomial Infection Surveillance System). Our previous studies suggested that these circulating strains were from a common pool [33]. For detailed studies of the antibiotic susceptibility patterns of CoNS in different growth modes, nine representative isolates were selected. These isolates included two biofilm-positive *S. epidermidis* (isolates 3 and 11), one biofilm-negative *S. epidermidis* (isolate 19), two biofilm-positive *Staphylococcus capitis* (isolates 8a and 9), two biofilm-weak/negative *S. capitis* (isolates 15 and 22), and two reference strains, *S. epidermidis* ATCC 35984 (RP62A) and *Staphylococcus hominis* ATCC 35982 (SP2), which were used as controls of biofilm-positive and biofilm-negative strains respectively.

**Table 1:** Bacterial isolates and growth media used for biofilm formation.

Isolate	Species	Status	**Growth medium for biofilm formation**^**a**^					
			**TSB**	**TSB+1% glucose**	**TSB+4% NaCl**	***icaA***	***icaC***	***icaD***
			
1	*S. warneri*	Invasive		+		-	-	-
2	*S. haemolyticus*	Invasive	-			-	-	-
3	*S. epidermidis*	Invasive	+			+	+	+
4	*S. epidermidis*	Invasive	+			-	-	-
5	*S. epidermidis*	Invasive	+			+	+	+
6	*S. capitis*	Invasive			+	+	+	-
7	*S. epidermidis*	Invasive	+			+	+	+
8a	*S. capitis*	Invasive			+	+	+	-
8b	*S. capitis*	Invasive			+	+	+	-
9	*S. capitis*	Invasive			+	+	+	-
10	*S. epidermidis*	Invasive		+		-	-	-
11	*S. epidermidis*	Invasive	+			+	+	+
12	*S. epidermidis*	Contaminant		w		-	-	-
13	*S. epidermidis*	Contaminant	+			+	+	+
15	*S. capitis*	Contaminant		w		+	+	-
16	*S. capitis*	Contaminant		+		+	+	-
17	*S. capitis*	Contaminant			+	+	+	-
18	*S. capitis*	Contaminant			+	+	+	-
19	*S. epidermidis*	Contaminant	-			-	-	-
20	*S. epidermidis*	Contaminant	+			+	+	+
21	*S. epidermidis*	Contaminant	+			+	+	+
22	*S. capitis*	Contaminant		w		+	+	+
23	*S. epidermidis*	Contaminant	+			+	+	+
24	*S. epidermidis*	Contaminant		+		+	+	+
RP62A	*S. epidermidis*	Reference	+					
SP2	*S. hominis*	Reference	-					

^a ^Selection of growth medium was based on the production of the highest biofilm density for each isolate. The amount of biofilm was indicated as: "+", strong (OD_600 _≥ 0.24); "w", weak (0.12≤ OD_600_ <0.24); "-", negative (OD_600_ <0.12). Isolates 8a and 8b were from the same baby, collected at different episodes of sepsis. Isolate 14 was not available for this study.

### Antibiotics

The antibiotics chosen for this study included those commonly used in NICUs for the management of neonatal sepsis; penicillin G (benzyl penicillin), gentamicin, vancomycin, and oxacillin. In the RWH NICU, the first line empiric treatment for early onset sepsis (first 48 h of life) is penicillin and gentamicin, and for late onset sepsis (>48 h), the choice is either flucloxacillin or vancomycin with gentamicin. Ciprofloxacin and rifampicin were also evaluated in this study as the efficacy of these antibiotics on CoNS biofilms has been reported by other in vitro studies. Penicillin G was purchased from CSL Biotherapies, Parkville, Australia and all others were obtained from Sigma-Aldrich, Castle Hill, Australia.

### Establishment of adherent monolayers

Bacterial cultures of adherent monolayers were established following the method of Miyake et al. (1992), which involved the addition of 50 μL volumes of bacterial suspension (1 × 10^6 ^CFU/ml in TSB) into wells of a 96-well flat-bottom microplate (Nunclon Delta; NUNC, Roskile, Denmark) [34]. The microplates were then centrifuged at 20°C for 10 min at 450 × *g*, followed by incubation at 35°C for 1 h. Non-adherent bacterial cells were removed by gently washing each well twice with 100 μL phosphate buffered saline (PBS, Amresco, Solon, Ohio, USA). The number of cells in adherent monolayers was determined by a series of steps: adding 100 μL PBS into each well, scraping the wells with sterile pipette tips, swabbing the wells with a cotton-tipped swab, mixing the contents, and pipetting 10 μL of the content for viable counts. The average bacterial count of four wells (column 12, row A-D) was used to represent all the monolayers in the microplates before any treatment.

### Establishment of mature biofilms

Biofilm bacterial culture was set up by a modification of an established method [35]. Briefly, a bacterial suspension of McFarland 0.5 (~ 10^8 ^CFU/ml) was diluted 1:100 into different diluents: TSB, TSB with 1% glucose or TSB with 4% NaCl, aiming to produce maximum amounts of biofilms for each individual isolate (Table 1) [33]. One hundred microlitres of the diluted bacterial suspensions were pipetted into each well in a 96-well flat-bottom microplate and incubated for 18-24 h at 35°C without shaking. After overnight incubation, the bacterial suspensions were aspirated and the wells were rinsed twice with 100 μL of PBS per well to remove non-adherent bacteria. After establishing biofilms in the 96-well microplate, the colony counts in four wells (column 12, rows A-D) were determined by the method of Cualtieri et al. (2006, 2007) and the mean was used to represent all biofilms in the microplates [36,37]. One hundred millilitres of Mueller-Hinton broth (MHB, Oxoid, Hampshire, England) was added into microwells containing biofilms. The biofilm matrix was then scraped with a sterile pipette tip, sonicated (Branson 450 digital sonifier with a microtip) for 8 s (4 × 2 s) at 10% of the maximum amplitude and viable counts were then performed.

### MICs for planktonic cells at mid-log phase and adherent monolayers

The MICs of single antibiotics for planktonic cells at mid-log phase were determined by standard broth microdilution [38]. To examine the MICs of single antibiotics for adherent monolayers, one hundred microliter volumes of twofold serial dilutions of antibiotics, ranging from 128 μg/ml to 0.001 μg/ml, in MHB or MHB plus 1% NaCl (for oxacillin) were added to microwells with established adherent monolayers. Plates were incubated at 35°C aerobically for 18 h (for all antibiotics except oxacillin and vancomycin) or 24 h (for oxacillin and vancomycin). MICs for both planktonic cells and adherent monolayers were defined as the lowest concentration of antibiotics that prevented the establishment of visible turbidity after overnight exposure.

### MBCs for planktonic cells at mid-log phase and adherent monolayers

The minimum bactericidal concentrations (MBCs) of the antibiotics against planktonic bacteria at mid-log phase were determined by a standard method (10). The log reduction for adherent monolayers was calculated by dividing the pre-determined viable count in the untreated wells by the viable counts in individual wells after antibiotic exposure. The monolayer MBC was defined as the lowest concentrations of antibiotics that reduced bacterial numbers by 3 logs.

### MICs for biofilm-grown bacteria

MICs using bacteria at stationary phase could not be tested by standard procedures as the maximum cell density had already been reached. The method to determine MICs for biofilm-grown bacteria was adapted from that of Ceri et al. (2001) [23]. Biofilms were established in 96-well microplates and exposed to 100 μL of twofold serial dilutions of antibiotics ranging from 1024 μg/ml to 0.001 μg/ml, in MHB or MHB plus 1% NaCl (for oxacillin). Microplates were then incubated at 35°C for 18 or 24 h, depending on the antibiotics tested. After overnight challenge, the supernatants from each well were carefully transferred to wells in a new 96-well microplate without disturbing the biofilms, and the turbidity of the contents was visually assessed. The biofilm MIC was defined as the lowest concentration of antibiotic at which no visible growth was observed. When exposed to antibiotics at or above this concentration, the planktonic bacterial population could not be established by bacterial shedding from the biofilms.

### MBCs for biofilms and planktonic cells at stationary phase

Determination of MBCs for the biofilms was also based on the reduction in viable counts after overnight exposure to antibiotics. After the MICs of the antibiotics for biofilms had been determined, the viable bacteria remaining in biofilms in wells with antibiotic concentrations above MICs were counted. MBCs for biofilms were calculated by dividing the pre-determined viable count of untreated biofilms by the viable counts of specified wells after antibiotic exposure. The MBCs of the antibiotics against planktonic bacteria at stationary phase were determined following the CLSI guidelines (2004) except that stationary phase cultures replaced log-phase cultures [38]. The MBCs for planktonic cells at stationary phase and for biofilms were defined as the minimum concentration of antibiotics required to reduce bacterial numbers by at least 3 logs.

### Effects of antibiotics at the highest achievable serum concentration on preformed biofilms

To determine the effects of conventional antibiotics on established biofilms, 100 μL volumes of TSB alone or of TSB containing the antibiotics at the highest serum achievable concentration (32 μg/ml for oxacillin, 32 μg/ml for vancomycin, 8 μg/ml for gentamicin) were added to established biofilms [39]. After incubation at 35°C for 24 h, the medium was aspirated from the wells and the biofilms were washed three times with PBS and then stained by 50 μl Hucker crystal violet (Merck, Kilsyth, Australia). Quantification of the biofilms was carried out by reading the absorbance OD_600 _as previously described [35]. Differences between the OD_600 _values of test wells and antibiotic-free wells were considered as an enhancement or reduction of the biofilms, depending on the trend of the change. After determination of antibiotic-bacteria combinations which showed increases in OD_600 _in spite of antibiotic challenge, epifluorescence microscopy and confocal laser scanning microscopy (CLSM) were performed to confirm the enhancement effect of antibiotics. In addition, a dose-response study was carried out for these combinations.

### Phase contrast microscopy, epifluorescence microscopy and confocal laser scanning microscopy

Phase contrast microscopy (Olympus 1 × 51, Hachioji, Japan) was used to examine the morphology of adherent monolayers formed by all 24 CoNS clinical isolates and two control isolates. Epifluorescence microscopy and confocal laser scanning microscopy was carried out to assess whether the increased amount of biofilm induced by antibiotic exposure was due to an increase in the amount of polysaccharide or bacterial cells, and to examine the viability of the biofilm cells after antibiotic challenge. After overnight exposure to the specific antibiotic, microwells containing CoNS biofilms were rinsed with 200 μl of 0.9% NaCl three times. Bacterial attachment and viability was then assayed with the LIVE/DEAD BacLight bacterial viability kit (Molecular Probes Inc., Eugene, OR), by adding 200 μl of a mixed solution of SYTO 9 (5 μM) (staining living cells) and propidium iodide (PI, 30 μM) (staining dead cells) into each well, and incubating the microplate at room temperature in the dark for 15 min. In parallel, 200 μl of a fluorescent dye specific for CoNS polysaccharide, Alexa Fluor 555 conjugated wheat germ agglutinin (10 μg/ml) (Molecular Probes Inc., Eugene, OR), were added to untreated and antibiotic treated biofilms, which were stained for 1 h in the dark. The biofilms were then examined by a Nikon Diaphot inverted fluorescence microscope equipped with B2 filter sets and G-2A filter sets, and by a Nikon Eclipse Ti inverted microscope equipped with Nikon A1R Fast Laser Scanning Confocal system. In CLSM experiments, samples were scanned frame-by-frame, at 488 nm to observe the live cells and at 561 nm for detection of dead cells and polysaccharide. A representative area in the centre of microwells was chosen for both epifluorescence microscopy and CLSM imaging.

### Data analysis

CLSI MIC experiments were repeated three times in triplicate. All other MIC and MBC experiments were repeated on three different occasions, and a fourth test was performed if the values were not identical. The geometric mean (log_2_) was calculated and used for comparison. Based on the acceptable range of MIC for antibiotic quality control for *Staphylococcus aureus* ATCC 25913, an increase of > 2 × log_2 _in MIC or MBC for oxacillin, vancomycin, ciprofloxacin and rifampicin, and an increase of > 3 × log_2 _for MIC or MBC for penicillin and gentamicin, were considered significant [38]. Experiments targeting the effects of antibiotics on preformed biofilms were repeated at least three times in triplicate. One way analysis of variance (ANOVA) or the non-parametric Mann-Whitney test was used for two-set comparisons and a *p*-value of < 0.05 was considered significant.

## Results

### Structure of adherent monolayers

The establishment of adherent monolayers of all 26 CoNS isolates after 1 h incubation in 96-well microplates was confirmed with 40 × 10 phase-contrast microscopy. A microcolony structure characteristic of biofilms was observed when incubation time was extended to 2 h for biofilm-positive CoNS.

### Susceptibilities of CoNS to conventional antibiotics

Most CoNS were resistant to penicillin G (100%), gentamicin (83%), and oxacillin (92%), and remained susceptible to vancomycin (100%), ciprofloxacin (100%) and rifampicin (79%). There was no significant difference in resistance profiles of invasive and contaminating strains (*p *≥ 0.05) (data not shown).

### Comparison of antibiotic susceptibilities of CoNS grown in different modes

#### Adherent monolayers compared with planktonic cultures at mid-log phase

In general, there were no significant differences in antibiotic susceptibility between planktonic cells at mid-log phase and adherent monolayers (determined by MIC and MBC). Two exceptions were: 1) MBCs of penicillin were 16 times higher for monolayer-grown *S. epidermidis* RP62A and *S. hominis* SP2 than planktonic cells, 2) MICs and MBCs of oxacillin were 8 times higher for monolayer-grown cells of isolate 8a than for planktonic cells (see underlined values in (Tables 2 and 3).

**Table 2:** Antibiotic susceptibilities of five biofilm-positive isolates^a ^grown in different modes.

Isolate and mode of growth	Initial bacterial density (CFU/ml)	Penicillin (μg/ml)		Gentamicin (μg/ml)		Oxacillin (μg/ml)		Vancomycin (μg/ml)		Ciprofloxacin (μg/ml)		Rifampicin (μg/ml)	
		
		MIC	MBC	MIC	MBC	MIC	MBC	MIC	MBC	MIC	MBC	MIC	MBC
**RP62A**													
Log Planktonic	(2.6-7.3)× 10^5^	8	8	16	32	64	64	2	2	0.06	0.12	0.008	0.03
Monolayer	(6.0-15.6) × 10^5^	32	128^c^	64	64	16	64	2	8	0.12	0.12	0.002	0.008
Stat Planktonic	(0.2-0.5) × 10^9^	^b^	> 1024		1024		> 1024		32		≤1		*32*
Biofilm	(0.9-1.9) × 10^9^	>**1024^e^**	>**1024**	64	>**1024**	64	>**1024**	**16**	>***1024***^***d***^	0.25	>***1024***	0.004	0.015
**Isolate 3**													
Log Planktonic	(2.9-8.3) × 10^5^	8	16	0.25	0.25	0.25	0.25	2	4	0.12	0.25	0.008	0.015
Monolayer	(5.0-11.0) × 10^5^	32	64	0.25	0.5	0.12	0.5	2	8	0.25	0.5	0.002	0.004
Stat Planktonic	(0.6-2.2) × 10^9^		>1024		32		*>1024*		*>*1024		*1024*		*256*
Biofilm	(0.3-1.3) × 10^9^	>**1024**	>**1024**	**16**	**16**	0.25	**2**	**16**	16	0.25	**2**	**0.5**	**4**
**Isolate 8a**													
Log Planktonic	(2.6-5.5) × 10^5^	128	128	32	32	8	8	1	2	0.25	0.25	0.008	0.03
Monolayer	(6.4-11.1) × 10^5^	>128	>128	32	64	64	64	1	8	0.12	0.5	0.004	0.015
Stat Planktonic	(4.4-4.9) × 10^8^		>1024		>1024		>1024		>1024		1024		2
Biofilm	(0.6-1.8) × 10^8^	1024	>**1024**	256	>**1024**	**256**	>**1024**	**8**	>**1024**	0.12	>**1024**	0.004	**4**
**Isolate 9**													
Log Planktonic	(3.1-5.2) × 10^5^	32	64	32	32	8	16	1	2	0.25	0.25	0.015	0.03
Monolayer	(4.6-10.8) × 10^5^	>128	>128	32	>128	16	32	2	8	0.25	0.5	0.004	0.015
Stat Planktonic	(0.7-0.8) × 10^9^		>1024		>1024		>1024		>1024		>1024		256
Biofilm	(1.1-1.7) × 10^9^	>**1024**	>**1024**	64	>**1024**	**>128**	>**1024**	**8**	>**1024**	0.25	>**1024**	0.008	**4**
**Isolate 11**													
Log Planktonic	(2.7-8.5) × 10^5^	8	8	64	>128	32	64	1	1	0.06	0.12	>128	>128
Monolayer	(3.2-17.8) × 10^5^	32	64	>128	>128	32	64	1	2	0.06	0.12	>128	>128
Stat Planktonic	(0.8-1.6) × 10^9^		>1024		>1024		>1024		>1024		512		1024
Biofilm	(0.2-0.6) × 10^9^	>**1024**	>**1024**	256	>**1024**	**256**	>**1024**	**8**	>**1024**	0.25	**16**	64	>1024

^α ^Biofilm-positive isolates (OD_600 _>0.24).

^b ^MICs for CoNS at stationary phase are not provided as the values could not be determined by standard methods.

^c ^Values underlined indicate a significant increase in the MICs or MBCs between log-planktonic and adherent monolayer modes of growth.

^d ^Values in italic indicate a significant change in the MBCs between stationary-planktonic and biofilm modes of growth.

^e ^Values in bold indicate a significant increase in MICs or MBCs between log-planktonic/adherent monolayer and biofilm modes of growth

**Table 3:** Antibiotic susceptibility of four biofilm-negative isolates^a ^grown in different modes.

Isolate and mode of growth	Initial bacterial density (CFU/ml)	Penicillin (μg/ml)		Gentamicin (μg/ml)		Oxacillin (μg/ml)		Vancomycin (μg/ml)		Ciprofloxacin (μg/ml)		Rifampicin (μg/ml)	
		
		MIC	MBC	MIC	MBC	MIC	MBC	MIC	MBC	MIC	MBC	MIC	MBC
**SP2**													
Log Planktonic	(1.2-4.1) × 10^5^	0. 5	0. 5	8	8	0.06	0.06	1	2	0.12	0.12	0.008	0.03
Monolayer	(2.6-4.7) × 10^5^	2	8^c^	16	16	0.12	0.12	0.5	1	0.12	0.12	0.004	0.008
Stat Planktonic	(3.5-9.5) × 10^7^	^b^	>1024		*1024*^d^		2		4		2		0.008
Biofilm	(1.0-3.2) × 10^7^	**128**	>**1024**^e^	8	32	0.12	**2**	2	4	0.12	**2**	0.002	0.008
**Isolate 15**													
Log Planktonic	(2.2-5.5) × 10^5^	128	128	32	64	16	32	4	4	0.25	0.25	0.015	0.03
Monolayer	(5.2-16.4) × 10^5^	>128	>128	32	64	16	64	4	8	0.12	0.25	0.004	0.008
Stat Planktonic	(3.7-7.9) × 10^8^		>1024		>1024		>1024		>1024		>1024		64
Biofilm	(0.7-0.9) × 10^8^	>**1024**	>**1024**	256	>**1024**	**256**	>**1024**	16	>**1024**	0.25	**512**	0.004	0.008
**Isolate 19**													
Log Planktonic	(2.9-7.4) × 10^5^	>128	>128	64	64	2	8	2	2	0.25	0.25	0.008	0.015
Monolayer	(3.2-14.6) × 10^5^	>128	>128	64	>128	2	8	2	8	0.12	0.25	0.002	0.008
Stat Planktonic	(0.7-1.5) × 10^9^		>1024		>1024		>1024		>1024		1024		2
Biofilm	(0.1-0.2) × 10^9^	1024	>1024	64	>**1024**	**64**	>**1024**	4	>**1024**	0.25	**4**	0.008	**0.06**
**Isolate 22**													
Log Planktonic	(2.8-5.6) × 10^5^	>128	>128	64	64	8	16	4	4	0.12	0.25	0.015	0.03
Monolayer	(4.8-16.3) × 10^5^	>128	>128	32	>128	32	32	2	8	0.12	0.25	0.004	0.008
Stat Planktonic	(0.5-0.7) × 10^9^		>1024		>1024		>1024		>1024		>*1024*		256
Biofilm	0.2 × 10^9^	**1024**	>1024	>128	>**1024**	**>128**	>**1024**	**16**	>**1024**	0.25	1	0.008	0.03

^a^ Biofilm-negative isolates included biofilm-weak producer (0.24 >OD_600_≥ 0.12) and biofilm-negative producer (OD_600_ <0.12).

^b ^MICs for CoNS at stationary phase are not provided as the values could not be determined by standard methods.

^c ^Values underlined indicate a significant increase in the MICs or MBCs between log-planktonic and adherent monolayer modes of growth.

^d ^Values in italics indicate a significant change in the MBCs between stationary planktonic and biofilm modes of growth.

^e ^Values in bold indicate a significant increase in MICs or MBCs between log-planktonic/adherent monolayer and biofilm modes of growth.

#### Biofilms compared with planktonic cultures at mid-log phase and adherent monolayers

Differences between susceptibilities of biofilm-grown cells and planktonic cells at mid-log phase were antibiotic dependent (Tables 2 and 3). The MICs of gentamicin, ciprofloxacin, and rifampicin were similar for most CoNS isolates studied. However, the MICs of three cell wall active antibiotics were significantly higher for biofilm cells than for planktonic cells at mid log-phase (32-256 times for penicillin G for 7 of 9 CoNS isolates, 8-32 times for oxacillin for 6 of 9 isolates, and 8 times for vancomycin for 5 of 9 isolates) (see bolded values in Tables 2 and 3). The MBCs of all antibiotics except rifampicin were significantly higher for isolates grown as biofilm cells than planktonic cells at mid-log phase. Five of nine isolates were equally susceptible to killing by rifampicin as biofilms and planktonic cells in mid-log phase. Adherent monolayers showed similar trends to planktonic cultures at mid-log phase (see bolded values in Tables 2 and 3).

#### Biofilms compared with planktonic cultures at stationary phase

The MBCs of most antibiotics were above the upper limit of detection (≥ 1024 μg/ml) for most CoNS isolates when grown as either planktonic cells at stationary phase or biofilms. For antibiotic/isolate combinations for which the MBCs could be compared between these two growth modes (n = 22), planktonic cells at stationary phase were either equally resistant (n = 7) or more resistant (n = 13) than biofilm cells (see italicised values in Table 2 and 3).

There was little difference between biofilm-positive and biofilm-negative phenotypes in their relative resistance to most antibiotics whether grown as planktonic cells, adherent monolayers or biofilms. However, most biofilm-positive, but few biofilm-negative isolates were more resistant to vancomycin (increased MICs) and rifampicin (increased MBC) in the biofilm mode than planktonic cells or adherent monolayers (Tables 2 and 3).

### Effects of antibiotics at the highest achievable serum concentration on preformed CoNS biofilms

**Figure 1:**
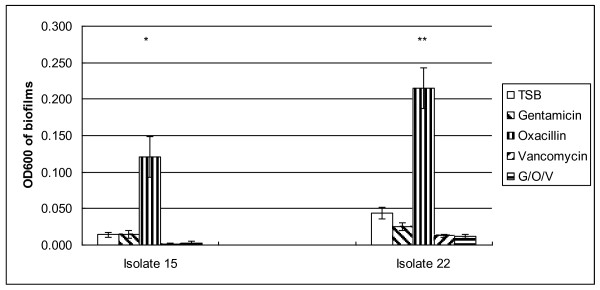
**Enhancement of biofilm-mode growth of CoNS cells by oxacillin at the highest serum achievable concentration.** Solutions of gentamicin, oxacillin, vancomycin or the three agents in combination in TSB at the highest concentrations achievable in serum, were added to biofilm grown cells of two *S. capitis* isolates; 15 (biofilm-negative, *ica*-weak), and 22 (biofilm-negative, *ica*-positive).  After overnight incubation, bacterial growth was stained with crystal violet and the OD_600_ was measured.  Error bars represent standard errors of at least 3 individual experiments in triplicate.  *: *P *= 0.004; ** *P* < 0.001.

**Figure 2:**
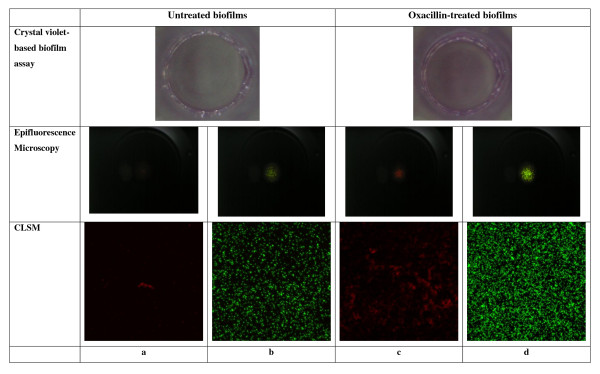
Enhancement of the biofilm growth of isolate 15 by oxacillin at the highest achievable serum concentration*  *: As isolate 15 and 22 showed similar responses, only the results for isolate 15 are shown.   a and c: stained with Alexa Fluor 555 conjugated wheat germ agglutinin; red signal indicates the presence of polysaccharide.  b and d: stained with  Live/Dead BacLight bacterial viability kit; green signal indicates the presence of live cells and red signal indicates the presence of dead cells.

The effects of oxacillin, gentamicin, vancomycin and their combination at the highest achievable serum concentrations on preformed biofilms of 24 CoNS clinical isolates are presented in Table 4. Vancomycin or combinations of vancomycin with oxacillin and gentamicin were the most effective in reducing the density of preformed biofilms (58.3%); followed by oxacillin (29.2%) and gentamicin (8.3%). Oxacillin stimulated biofilm growth of two *S. capitis* isolates (isolates 15 and 22), converting these biofilm-negative isolates (OD_600 _< 0.12, when grown with TSB) into weak biofilm producers (0.12 ≤ OD_600 _< 0.24) or even biofilm-positive phenotypes (OD_600 _≥ 0.24) (Figure 1 and Figure 2). *S. epidermidis* isolate 19 also showed enhancement of biofilm in the presence of oxacillin but the difference did not quite reach clinical significance (*p *< 0.074). Similar findings were obtained when 1% glucose was added into TSB as the medium for biofilm growth (data not shown). Dose-response studies using isolates 15 and 22 confirmed that oxacillin at concentrations ranging from 0.25 to128 μg/ml enhanced the biofilm-mode growth, with peak enhancement occurring around 8 to 32 μg/ml (data not shown).

**Table 4:** No. of CoNS isolates showing reduction^a^ or enhancement when biofilms/adherent cells exposed to antibiotics.

Effect	Number (%) of isolates affected by			
	
	Gentamicin (GEN)	Oxacillin (OXA)	Vancomycin (VAN)	GEN, OXA, VAN
Reduction	2 (8)	7 (29)	14 (64)	14 (64)
Enhancement	0 (0)	2 (8)	0 (0)	0 (0)
None	22 (92)	15 (63)	10 (36)	10 (36)

^a ^After overnight antibiotic exposure, the difference in OD_600 _values between test biofilms and antibiotic-free wells, if greater than intra-microplate variability, were defined as significant; a decrease was considered a reduction and an increase was considered an enhancement.

### Microscopic analysis of the CoNS biofilm enhancement by oxacillin

In crystal violet staining, epifluorescence microscopy, and CLSM imaging experiments, exposure to oxacillin at 32 μg/mL increased the thickness of biofilms of both isolates 15 and 22. Figure 2 show a representative field of view obtained for isolate 15. In CLSM imaging experiment, the Live/Dead BacLight bacterial viability kit and Alexa Fluor 555 conjugated wheat germ agglutinin were applied to the biofilm formed by isolates 15 and 22 before and after exposure to oxacillin at 32 μg/ml. For both isolates, oxacillin stimulated the production of polysaccharide (Figures 2a and 2c), and the multiplication of living cells attached to the polystyrene surface (Figures 2b and 2d).

## Dicussion

The prevalence of antibiotic resistance of CoNS isolated from VLBW infants in the NICU at RWH, was similar to that of a previous study (100%, 93%, 80%, 4% and 0% resistant to ampicillin, methicillin, gentamicin, ciprofloxacin and vancomycin respectively) [40]. Invasive isolates and contaminants exhibited similar susceptibilities. The same groups of invasive and contaminating isolates showed no differences in biofilm production or possession of the *ica *genes [33], suggesting that resistant strains were acquired initially as skin flora and subsequently caused invasive infections in susceptible infants.

This is the most comprehensive study so far to compare antibiotic susceptibilities of bacteria in the whole range of growth modes relevant to biofilm resistance. Planktonic cultures at mid-log phase, adherent monolayers, mature biofilms and planktonic cultures at stationary phase were assessed for their susceptibilities to multiple antibiotics. The rationale was to challenge bacterial cultures at different stages and modes of growth with the same amount of antibiotic. In particular, we wanted to compare the susceptibilities of biofilm-grown cells and planktonic cells of the same cell density.

The nine representative strains used in our study generally showed similar changes in antibiotic resistance in response to changes of growth modes, regardless of their different species and sources. In agreement with the study by Labthavikul et al.(2003), we found that MICs and MBCs were generally similar when CoNS were grown in the planktonic mode or as adherent monolayers [41]. The few exceptions were three isolates that were more resistant in the adherent monolayer form to β-lactam antibiotics; penicillin and oxacillin. Other studies found that *S. aureus*, *Escherichia coli* and *Pseudomonas aeruginosa* were significantly more resistant to both growth inhibition and killing in the adherent form than in the planktonic form [19,34]. This difference could possibly be explained by different responses to antibiotics by individual species [19]. Different methods used for monolayer formation might also be responsible for the different findings from these studies. Miyake and colleagues (1992) challenged *S. aureus* monolayers after 1 h incubation while Aaron et al. challenged 2 h monolayers of *P. aeruginosa*[19,34]. In our preliminary study, we found that most biofilm-positive CoNS formed microcolonies after incubation for 2 h. Microcolonies represent early stage of biofilm formation and demonstrate some properties of biofilms [42]. Thus it is possible that the study of Aaron et al. (2002) examined early-stage biofilms rather than adherent monolayers, however, it is also likely that the difference was due to the different kinetics of biofilm formation between *P. aeruginosa* and *Staphylococcus* spp. [19].

Conventional MICs have been used to guide the treatment of biofilm-related infections at the febrile stage, based on the assumption that biofilm-released cells are similar in their susceptibilities to cells in the planktonic phase [23]. Reported differences between the conventional MICs and biofilm MICs were attributed to lack of standardization of initial inocula and to the presence of small colony variants [23]. However, in our study, we found that MICs of cell wall active antibiotics were frequently higher for biofilm grown bacteria than planktonic cultures. This is consistent with recent studies by Moskowitz et al. (2004) and Melchior et al. (2006), who reported that biofilm MICs of β-lactam antibiotics, but not other antibiotics, were much higher than the conventional MICs for *P. aeruginosa* and *S. aureus* respectively [21,24]. These results are not surprising given that cell wall active antibiotics mainly affect rapidly growing bacteria. Biofilm-released cells are likely to be less active than dividing planktonic cells at mid-log phase, probably because they have recently undergone a switch from a biofilm mode of growth to a free-living mode, similar to cells at lag-phase growth, and require a change in gene expression to adapt to the new environment.

The MBCs of penicillin G, gentamicin, oxacillin, and vancomycin for CoNS grown in a biofilm mode were generally > 1024 μg/ml, which is well beyond the highest achievable serum concentrations. Although some of these antibiotics at the highest achievable serum concentrations were effective against bacteria grown planktonically to mid-log phase, they were inadequate to kill bacterial biofilms. Administration of a single antibiotic to newborns with catheter-associated infections, based on the results of in vitro susceptibility tests designed for planktonic bacteria at mid-log phase, is unlikely to reach an effective concentration to eradicate the bacteria adherent to the catheters. This may be one of the reasons that explain the frequent failure of treating CoNS infections with conventional antibiotics in patients with foreign body infections when the devices are not removed.

The MBCs of rifampicin and ciprofloxacin were generally higher for planktonic cells in stationary phase than their biofilm-grown counterparts. This is consistent with the finding of Spoering and Lewis (2001), that stationary phase cultures showed similar or higher resistance than biofilm-grown bacteria [17].

A possible limitation of this study was the assessment of killing of biofilm bacteria using the biofilm MBC, which leaves 0.1% of the challenged cells out of our consideration. This remaining 0.1% population might contain "persister cells", which have been considered as an important mechanism of biofilm resistance [17]. While the endpoint of complete killing may be more desirable, MBECs of the first-line antibiotics used in NICUs were far above the upper detection limit used in this study (1024 μg/ml) [29].

An important aim of this study was to investigate the effect of antibiotics on the various stages of biofilm development. On initial attachment to a polymer surface, CoNS may be susceptible to antibiotics, but resistance increases as the biofilm develops. These findings highlight the importance of early recognition and treatment of catheter-related neonatal sepsis. At the early attachment stage success is possible with the widely used first-line antibiotics guided by conventional susceptibility tests. It should be noted that some CoNS are more resistant to β-lactam antibiotics in the adherent monolayer phase. Once biofilms have developed, cell-wall active antibiotics often fail to resolve the febrile phase of the infection, as these antibiotics at CLSI MIC cannot reliably prevent biofilms from releasing free-living cells. Moreover, most antibiotics, with the exception of rifampicin, are incapable of killing CoNS within the biofilm.

No significant difference was found in the response to antibiotics between biofilm-negative and biofilm-positive phenotypes. However, it is important to note that isolates classified as biofilm-negative (OD_600 _< 0.12), if grown under biofilm assay conditions, do produce densely-grown microcolonies [43]. This information, together with our finding that there was little difference in susceptibility between biofilm-grown bacteria and planktonic-grown bacteria of the same density, suggests that a densely adherent growth mode is an important determinants of biofilm resistance. Although this finding is contrary to the current belief that higher biofilm-forming ability is associated with antibiotic resistance [42,44], it is supported by studies that reported no correlation between the amount of slime produced and antibiotic susceptibility of *S. epidermidis*[41,45]. Moreover, we have reported a similar level of resistance of CoNS isolates to antibiotic lock solutions, regardless of the amount of biofilm produced [29].

To our knowledge, this is the first study to investigate whether commonly used antibiotics at the highest achievable serum concentrations are able to stimulate the growth of established biofilms. It is also the first study which visually demonstrates the enhancing effect of external stimuli on two principal components of biofilms; polysaccharide and biofilm bacteria. Other studies focused on the effect of antibiotics at subinhibitory concentrations on development of biofilms [31,46]. Rachid et al. (2000) showed that oxacillin at subinhibitory concentration did not induce the expression of *ica *operon and biofilm formation [31]. Different subspecies/strains and concentration of oxacillin used by these investigators might explain the lack of biofilm enhancement by oxacillin. Cerca et al. (2005) reported that exposure to subinhibitory dicloxacillin, an antibiotics structurally similar to oxacillin, could stimulate the polysaccharide synthesis in *S. haemolytics*[30]. In this study, oxacillin at the highest achievable serum concentration (32 μg/ml) enhanced biofilm growth of two biofilm-negative *S*. *capitis *(both *ica *< positive, oxacillin MIC < 16 μg/ml). Biofilm enhancement in both *S*. *capitis* isolates was related to an increase of bacterial numbers and in the amount of polysaccharide produced. The mechanism of oxacillin enhancing biofilm formation of *S*. *capitis *still requires further investigation. Biofilm production in *S*. *capitis *can be induced by external stresses such as a high NaCl level through induction of the expression of *ica *locus by repression of tricarboxylic acid cycle [47,48]. It is likely that the positive influence of oxacillin on biofilm production demonstrated in this study is also via the stress-induced *ica *expression. The enhancement of biofilm production by oxacillin might explain some treatment failures, as flucloxacillin is frequently used to treat infections caused by methicillin-sensitive CoNS.

## Conclusion

In summary, our study confirmed previous findings that CoNS in a biofilm mode of growth are highly resistant to most antibiotics. Most first-line antibiotics used in NICU, at an achievable serum concentration, failed to eradicate the CoNS biofilms. Only rifampicin and, in some cases ciprofloxacin, consistently displayed activity against biofilm-grown bacteria. Our results also indicated that either a high-density growth phase or its metabolic state, which is related to a biofilm growth mode rather than biofilm-producing ability, contribute significantly to the resistance of CoNS biofilms to antibiotics. Finally, we have cited examples of the enhancement of CoNS biofilms by clinically achievable concentrations oxacillin, representing the commonly used β-lactam resistant penicillins.

## Competing interests

The authors declare that they have no competing interests.

## Authors' contributions

MD, YQ, TI, AD, SG designed the study. YQ performed laboratory work, drafted the manuscript. MD, TI, AD and YQ edited the manuscript. All authors read and approved the final manuscript.
